# Supplementary material for: An experimental approach to training mood for resilience
Source: PLoS One. 2023 Sep 7;18(9):e0290881. doi: 10.1371/journal.pone.0290881 (PMC10484456; doi:10.1371/journal.pone.0290881)

Screenshots of the task


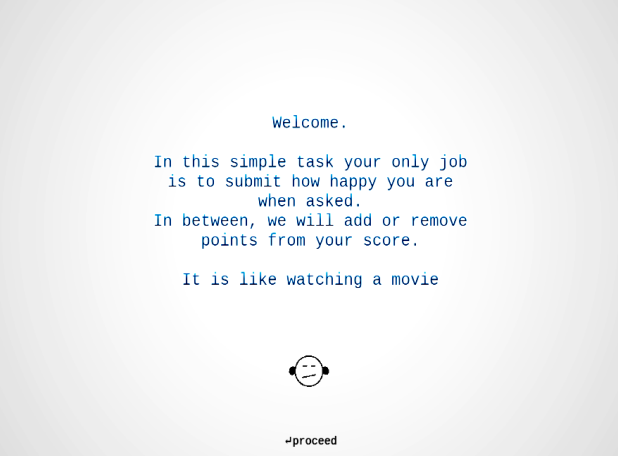

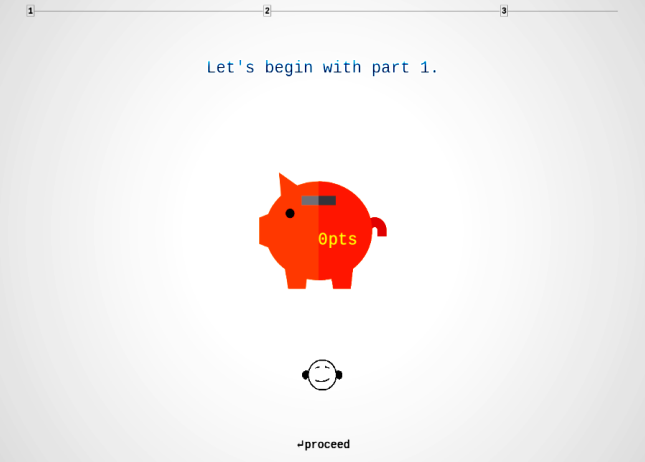


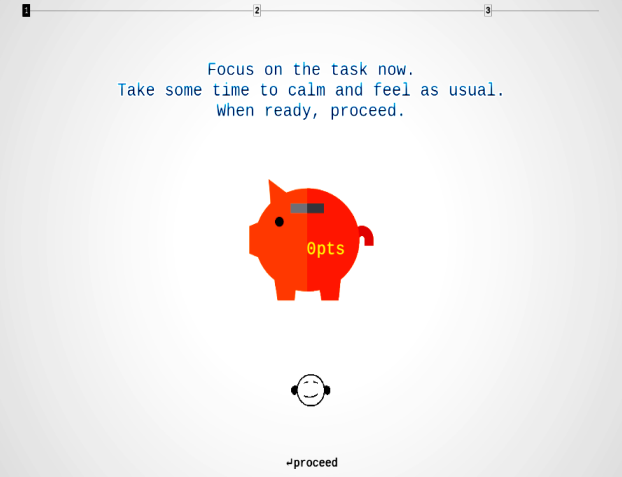

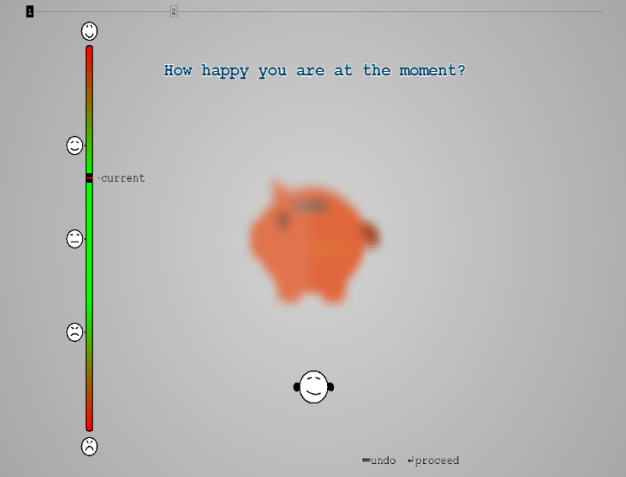


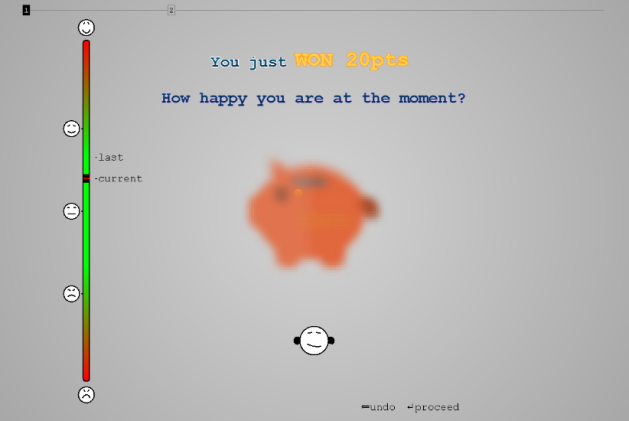


Retest mood ratings for the five environments where mood training was successful and promoted resilience

| Reverse-proportional reward of good mood  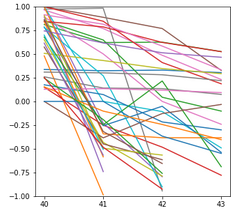 | Constant medium reward of good mood  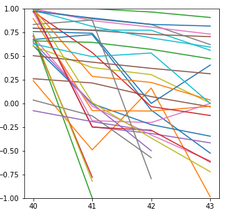 |
| --- | --- |
| Constant maximum reward of good mood  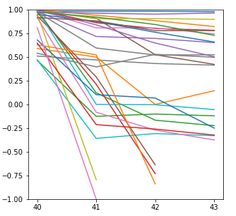 | Constant maximum reward of bad mood  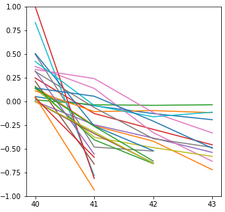 |
| Reverse-proportional reward of bad mood  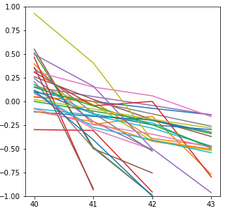 | Control group  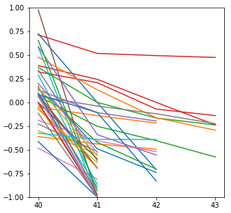 |

|  | Environment | Filter A | Filter B |
| --- | --- | --- | --- |
| Study 2 | proportional reward of good mood | 10 | 8 |
|  | reverse-proportional reward of good mood | 9 | 9 |
|  | medium reward of good mood | 7 | 8 |
|  | maximum reward of good mood | 7 | 6 |
|  | proportional punishment of good mood | 18 | 12 |
|  | reverse-proportional punishment of good mood | 10 | 7 |
|  | medium punishment of good mood | 8 | 7 |
|  | maximum punishment of good mood | 8 | 7 |
|  | proportional punishment of bad mood | 10 | 11 |
|  | reverse-proportional punishment of bad mood | 10 | 9 |
|  | medium punishment of bad mood | 9 | 10 |
|  | maximum punishment of bad mood | 11 | 15 |
|  | proportional reward of bad mod | 7 | 8 |
|  | reverse-proportional reward of bad mod | 8 | 7 |
|  | medium reward of bad mood | 9 | 8 |
|  | maximum reward of bad mood | 6 | 6 |
| Study 3 | reverse-proportional reward of good mood, 10 rounds | 7 | 6 |
|  | maximum reward of good mood, 10 rounds | 8 | 7 |
|  | reverse-proportional reward of good mood, 20 rounds | 15 | 10 |
|  | maximum reward of good mood, 20 rounds | 13 | 11 |

Table 1. The number of participants excluded from each experiment based on our filters for participant selection are presented here. Filter A corresponds to mood responsiveness to three specific, consecutive stimuli (see section Filters for participant selection for the second set of experiments)*.* For Filter B participants were excluded from the task if during the test or retest sequence the last registered mood was higher than the mood before the beginning of the test or retest sequence.

Further Analyses

Mixed Effects Model for evaluating the influence of task rewards on mood

In addition to our linear regression model, a mixed effects model was created that links the effect of task stimuli to mood, for each task round, using the participants as a random variable. The model showed that the task stimuli had a statistically significant effect on mood (Ν_obervations_= 6560, N_subjects_=164, β=1.183, SE=0.053, p<0.000001).

mood_change_n ~ stimulus_n + (1+stimulus_n|subject)

stimulus_n: stimulus that is administered in round n

mood_change_n: the difference in mood (mood_n_-mood_n-1_) after the administration of stimulus_n

Task Reliability

To examine task reliability, we looked at how mood responds to task stimuli, at different time points of the task. Specifically, we separated the task rounds in two groups with odd and even task round numbers, respectively. We run the mixed effects model created above to examine whether the task round groups would impact mood’s response to stimuli. The model showed that the task stimuli had a statistically significant effect on mood (Ν_obervations_= 6560, N_subjects_=164, β=1.183, SE=0.053, p<0.000001), irrespective of the task round group (odd or even).

mood_change_n ~ stimulus_n + group_type + (1+stimulus_n|subject)

group_type: odd(1) or even(0) task round number.

Sensitivity Analysis for the test sequence’s number of stumuli

To examine whether our results around mood training would change depending on the number of stimuli of the test and retest sequence, we repeated our analyses for Study 3, using the first or the first and second of the three stimuli of the test and retest sequence. Our results did not change when less than three stimuli were included in the test and retest sequence.

Study 3 consists of four experiments, changing the length of the test and retest sequence, in each of them-a total of eight analyses-did not alter the original results.

Test and retest sequences with one stimulus, 10 rounds of the pseudorandom environment

| Environment | β-coef | CI(2.5,97.5) | SE | p value |
| --- | --- | --- | --- | --- |
| reverse-proportional reward of good mood | 0.177 | 0.013, 0.341 | 0.082 | 0.034 |
| **maximum reward of good mood** | **0.245** | **0.089, 0.401** | **0.077** | **0.006** |

Test and retest sequences with two stimuli, 10 rounds of the pseudorandom environment

| Environment | β-coef | CI(2.5,97.5) | SE | p value |
| --- | --- | --- | --- | --- |
| reverse-proportional reward of good mood | 0.175 | 0.007, 0.343 | 0.083 | 0.041 |
| **maximum reward of good mood** | **0.228** | **0.067, 0.388** | **0.080** | **0.006** |

Test and retest sequences with one stimulus, 20 rounds of the pseudorandom environment

| Environment | β-coef | CI(2.5,97.5) | SE | p value |
| --- | --- | --- | --- | --- |
| reverse-proportional reward of good mood | 0.110 | -0.028, 0.249 | 0.069 | 0.115 |
| **maximum reward of good mood** | **0.304** | **0.172, 0.436** | **0.066** | **0.0000** |

Test and retest sequences with two stimuli, 20 rounds of the pseudorandom environment

| Environment | β-coef | CI(2.5,97.5) | SE | p value |
| --- | --- | --- | --- | --- |
| reverse-proportional reward of good mood | 0.118 | -0.025, 0.260 | 0.071 | 0.102 |
| **maximum reward of good mood** | **0.307** | **0.170, 0.442** | **0.068** | **0.0000** |

Sensitivity Analysis for the last experiment

To determine the magnitude of effect we were powered to detect in our last survived experiment (“maximum reward of good mood, after 20 rounds”), a sensitivity alalysis was conducted.

For this experiment with 121 participants (59 experimental and 62 controls), 3 predictors and alpha of 0.0166 after Bonferoni correction (for 3 terms), we had 80% power to detect an f^2^ of 0.0743.


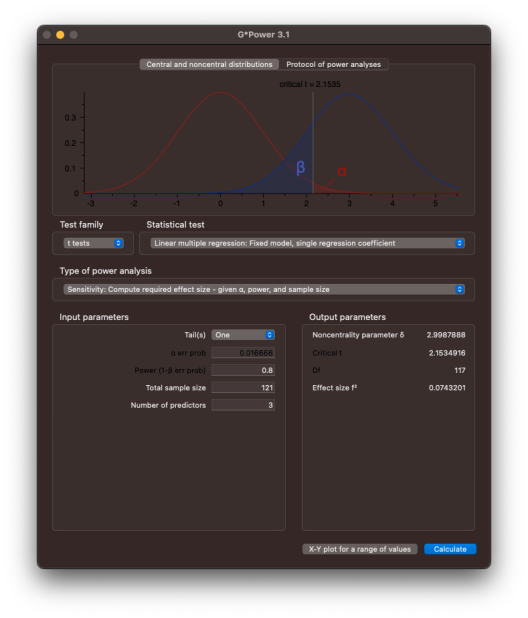

Supplement: S1 File — (DOCX) [file pone.0290881.s001.docx]
