# Supplementary material for: An experimental approach to training mood for resilience
Source: PLoS One. 2023 Sep 7;18(9):e0290881. doi: 10.1371/journal.pone.0290881 (PMC10484456; doi:10.1371/journal.pone.0290881)
Supplement: Attachment — Submitted filename: Reviewers-replies.docx [file pone.0290881.s002.docx]

Dear editor,

Please find below our our replies to your concerns as well as the rebuttal letter addressing the reviewers’ comments for our manuscript.

We thank you for your comments on our manuscript. We have implemented the following changes as per your suggestions:

- Concerning the power of our analysis, we have now addressed that in our replies to the reviewers and have included a sensitivity analysis in the Supplementary Material.
- We have amended our introduction to make it more concise and avoid redundancy.
- Proof-read the manuscript and corrected any mistakes. We have also made several changes in our sentence-structure and punctuation to make it clearer and easy to follow.
- Concerning the term “mood resilience”, in our introduction we define mood resilience (see below) and we explain that we refer to it in relation to exposure to negative stimuli. In order to be more precise, however, we have now replaced, in our manuscript, “mood resilience” with “mood resilience to negative stimuli”, when possible.

“The term resilience refers to the capacity of a dynamic system to withstand or recover from significant disturbances that threaten its adaptive function, variability or development. In relation to mood and its interaction with the environment, mood resilience could be viewed as the ability of mood to resist the effects of exposure to adverse stimuli or bounce back from states provoked by such an exposure. For the purposes of our study, and concerning resilience, we focus on the first component of the definition and explore whether mood could be trained to resist exposure to negative stimuli.

Our rebuttal letter to the reviewers follows.

Sincerely,

Vasilios Mantas, MD, PhD

Vasileia Kotoula, PhD

Dear reviewers,

Thank you very much for carefully reading our manuscript and for all your comments and suggestions which we have tried to address, to the best of our ability.

**Reviewer 1**

*The goal of this study was to test a method for training mood resilience, namely a way of decreasing mood sensitivity to negative stimuli such as losses in a gambling task. To this end, a series of experiments were conducted to first generate and then confirm hypotheses regarding the respective impacts of different task environments. The authors argue that this type of basic research may offer a future avenue for training mood and enhancing well-being.
They present a methodologically sound experimental paper and a well-written manuscript. The number of sub-experiments and progression from exploratory to confirmatory point towards a rigorous research process.
I have only minor suggestions to polish an already good manuscript.

Introduction:
- Good review of previous studies in the field and clear mention of what the current study adds. The research question is clearly stated and the reasoning that leads up to the hypotheses seems sound.

Method:
- Overall, the methods section could benefit from another proof-reading (including punctuation and sentence structure), as it contains a few sentences which are difficult to understand. However, I appreciate the clear statements on what was expected for each experiment/analysis, as it makes following the experiments easier.*

We thank the reviewer for their positive comments on our Introduction and Methods sections. Following their suggestion, we have now proof-read the Introduction and have made several changes and corrections to our sentence structure and punctuation. We hope that this makes our Methods section clearer and easy to follow.

*- On page 5, it is stated “administration of task stimulus lasted approximately 3 seconds”: What does “approximately” mean in this context, did this time window differ between experiments/participants/…?*

The word “approximately” is used in our manuscript to account for the minor differences that could have occurred during the stimuli administration, as the task was executed online, via Amazon MTurk, on each participants’ personal device – this makes the exact timing of the stimulus dependant on the characteristics and condition of each machine. As those timing differences would have been very small (within the milliseconds range), we do not expect that they could have impacted the results of our experiments.

*- Marker for “last” on the mood-o-meter (p. 7): The manuscript states this was added to help participants focus on their momentary mood, compared to their previously registered mood. Could this not also induce expectancy effects, e.g. if participants assume they should feel better after a reward, but wouldn’t actually indicate a better mood without the marker of their previous response?*

Thank you. As we state in our manuscript, the participants’ previously registered mood value was marked as “last” on the meter “to help participants on their judgement of momentary mood in relation to their previously registered mood”. This helps participants to have a better estimate of whether their momentary mood is better or worse, after winning or losing points, in relation to their previous mood. We believe that when the previous momentary mood value is marked on the meter, there is less variability, within subjects, between mood ratings corresponding to similar momentary mood states. In contrast to that, if no indication about the previous mood is used, and as the subjects might not remember the exact position of their previous rating, it is possible that when they are happier, for example, they could choose a lower meter value. Although in both cases, using the marker or not, expectancy effects could be present, we cannot exclude the possibility that our manipulation could have enhanced them. In order to be able to properly address this question, in future studies, a new experiment could be conducted to specifically test for this.

We have now updated our limitations section to also include this point and the relevant section reads as follows:

“Concerning our task design, the word “last” was shown on the mood-o-meter to help participants better assess their momentary mood, in relation to the previously inputted mood value. It is possible, however, that this indication could have further enhanced or somehow impacted expectancy effects, influencing participants into mood rantings strongly based on the previous stimulus.”

*- Participants: Was there a maximum age allowed to enter? Are any other demographic data available for the sample? Did the authors conduct a power analysis, and if not, what is the reasoning and how else did they arrive at this sample size (i.e., how was determined when to stop data collection)? How was the distribution between studies decided, and more importantly, how was the distribution between task environments within a study decided (were participants randomized or what determined their allocation)?*

We thank the reviewer for these comments.

We did not impose a maximum age limit for our experiments. The only additional demographic data that are available and not already mentioned in our manuscript concern the fact that our participants were located in the USA. This has now been added in our Methods.

Concerning the power analysis, the second set of our experiments, as mentioned in our manuscript, is an exploratory one and a power calculation was therefore not conducted. For those experiments, we did not focus on studying the task environments *per se,* but rather on the fact that mood could be trained. As a result, we aimed for a statistically significant effect that could be visible even with a relatively small sample size. Given the risk of small sample sizes -mainly type II error but also a risk for type I error (Leppink et al., 2016)- , we replicated our findings (for the first two most effective environments), in the next steps of our study, in a larger sample, and also examined whether this effect could survive over time. Since a power calculation was not conducted for that experiment and a post-hoc power analysis is not considered a valid method to assess power (Dziak et al., 2020), we instead conducted a sensitivity analysis to determine the magnitude of effect we were powered to detect (we have added information about this sensitivity analysis to supplementary material). For the last replication experiment with 121 participants (59 experimental and 62 controls), 3 predictors and alpha of 0.0166 after Bonferroni correction (for 3 terms), we had 80% power to detect an f2 of 0.0743. Since we have been careful not to base our conclusions on the lack of significant results, the primary threat to our interpretation from an underpowered study would be type-s errors (Gelman and Carlin, 2014). An f2 of 0.0743 is considered small (Cohen, 1998; Selya et al., 2012) and reasonable to observe for an intervention modifying the response of mood. As such, we do not think a type-s error is likely. Additionally, the statistical power of that analysis is also reflected on the confidence intervals (CIs) of our results (Hoenig and Heisey, 2001; Schulz and Grimes, 2005; Vandenbroucke, 2007), in the task environment which yielded positive results and survived Bonferroni correction. To summarize, the main question addressed in our manuscript is whether mood can be trained which means that one positive finding is sufficient. As such, for the environment that had the largest effect, two more repetitions were performed in novel and larger samples that yielded statistically significant results. Moreover a sensitivity analysis for the final experiment was conducted which showed that it had the power to detect a small effect size. Given that, a type-I or type-s error seems unlikely. Since our main question or interpretation is not based on statistically non-significant results type-II errors, although possible for the remaining environments, do not affect our study.Our sensitivity analysis is now included in our Supplementary Material.

Concerning the distribution of participants between experiments (task environments), each participant could participate in any of these experiments only once. Meaning that no participant has participated in more than one study or more than one task environment within a study. This was secured by giving each subject who accessed our task a “qualification”, that is a function that allows us to mark this participants and exclude them from future experiments. The MTurk participant pool is large enough to allow us to have each experiment performed by different subjects. Each of our studies was uploaded as one experiment -one single hit- and was completed before the next experiment was uploaded.

*Results
- Both the analysis and results sections seem very detailed to me and contain clear explanations as to why certain steps were or were not taken (e.g., why exploratory analyses were not corrected for multiple testing whereas confirmatory analyses were indeed corrected).
- It is surprising to me that such high percentages (80% and 91% in study 1) of participants showed mood responsiveness to a relatively simple reward/punishment task (although this might be because it is not my field of expertise). If I understood it correctly, the criterion for classifying participants as non-responsive was if they gave the same rating three times in a row. How much did ratings fluctuate (e.g., range of ratings)? And do the authors have any hypotheses for why some individuals might be more responsive to their task than others?*

We thank the reviewer for their comments on our Results’ section.

Indeed, as the reviewer mentions, in our experiments, three consecutive rounds with large reward differences, that were identical for all participants were used to filter out those participants whose mood was not responsive to the task stimuli. We have now calculated, as requested by the reviewer, the range of ratings between participants in these three rounds (n: 187, max: 2, min: 0, mean: 0.56, median: 0.45, std: 0.44). We would like to point out, however, that participants were not only excluded when giving the same mood ratings for all three stimuli but rather when their ratings did not follow the stimuli fluctuations. Examples of this include, participants inputting a smaller mood value, compared to their previous rating, for a stimulus that has a much larger value than the one administered before, but also them giving the same mood rating for all stimuli. For example, when the next stimulus is greater than the one before which includes, of course, them giving the same rating in all three stimuli.

We do not have any evidence suggesting that participants in our experiments are more responsive, compared to other, similar tasks. However, the fact that our task was a JavaScript task that run smoothly and had pleasing visual elements, may have increased the participants’ engagement leading to more accurate responses. Concerning the high percentages of participants that showed mood responsiveness within our experiment, there could be several reasons as to why some individuals might be more responsive to our task, compared to others. This could be due to interindividual systemic differences that could have a neurobiological and/or neurochemical basis (trait) (Laricchiuta & Petrosini, 2014), as well as be attributed to the specific moment in time when our experiment was conducted. For example, positive or negative experiences that a participant could have had on that specific day, would influence how they rate their mood in response to our task stimuli (state) (Neumann et al., 2001). Finally, it is also possible that some participants were not paying attention to the task but only aiming for the final compensation (non compliant subjects).

*Discussion:
- The authors offer a critical discussion of their results, including study limitations, without diving into interpretations that would not be suitable to the experimental set-ups. Similar to the introduction, the discussion section benefits from clear reasoning and is easy to follow. I also appreciate that implications are discussed mostly in relation to future research, seeing as clinical implications of such basic research would not be appropriate.
- Even though the manuscript already contains discussion of several limitations, I would recommend adding an additional 1-2 sentences on generalizability of the results considering this particular sample.*

We thank the reviewer for their positive comments. Given the conflicting nature of the literature around MTurk samples (see Levay et al., 2016; Litman and Robinson, 2021 but also Walters et al., 2018) and the fact that we, ourselves, did not collect detailed sociodemographic information, we have commented, in our limitations, around the generalizability of our results as this is limited by our sample.

“Additionally, since this is an online sample, rather than a random sample from the general population, we caution on inferences about the generalizability of our findings.”

**Reviewer 2**

*The aim of the present exploratory study was to examine whether mood can be trained to become more resilient to future negative events. The authors developed a new series of experiments, in which participants rated their current mood state when rewards or punishments were administered. In the first series of experiments, the task was validated. In the second and third series of experiments, the authors examined which different task environments are efficient in training mood to become more resilient to negative stimuli.

The approach taken is innovative and the results provide an interesting starting point for future research that aims to examine the effectiveness of mood training in healthy and clinical populations including individuals with depression. Below are a number of minor and major points that should be addressed prior to publication:

Abstract:
-Please be more concise regarding the methods and results of the current study. For example, please clearly describe which environments were found to be effective. Please e.g. also clearly state that this was an online study conducted in healthy volunteers (including information on the number of participants).*

We thank the reviewer for their comments on our abstract, we have now tried to amend the abstract as per their suggestions and the relevant changes are included below:

“For this study, a new task was developed where participants register their mood when rewards are added or subtracted from their score. The study was conducted online, using Amazon MTurk, and a total of N=1287 participants were recruited for all three sets of experiments.

In an exploratory experiment, sixteen different experimental task environments which are characterized by different mood-reward relationships, were tested.

We identified six task environments that produce the greatest improvements in mood resilience, as measured by decreased sensitivity to loss. In a next step, we isolated the two most effective task environments, from the previous set of experiments, and we replicated our results and tested mood’s resilience over time, in a novel sample. We found that the effects of the task environments on mood are detectable and remain significant after multiple task rounds (approximately two minutes) for an environment where good mood yielded maximum reward.”

*Introduction:
-The introduction should be shortened. For example, there is some redundancy in the description of the different series of experiments on page 4.*

We thank the reviewer for their suggestion, we have now tried to make our Introduction more concise. Given, however, the exploratory nature of our experiments and the novelty of our task, some repetition is unavoidable in order to facilitate understanding of the manuscript even by non-expert readers.

*Methods:
-The question: !How happy are you at the moment?” is not neutral. Why did the authors not use a more neutral question regarding current mood?*

We thank the reviewer for this comment. Our use of the question “How happy are you at the moment?” is in line with previous computational experiments that have used the mood-o-meter to measure momentary mood in response to positive and negative stimuli (for example: Keren et al., 2021; Rutledge et al., 2014) . As these experiments are considered effective in capturing momentary mood, we did not wish to deviate from their methodology. We do recognize, however, that the “positive” phrasing of the question could bear the potential for positive bias. In order to investigate that, differently phrased questions can be asked, in future experiments, to establish the effects of exact phrasing.

*Participants:
-Very little sociodemographic information is provided (e.g. SES). Therefore, the generalizability of the findings remains unclear. This should be stated as a limitation. What was the age range of participants (18 to ?).
How exactly did the authors ensure that participants only participated once? Was the IP address tracked?*

Extensive sociodemographic data were not collected for this study. The only information that is available and was not already mentioned in our manuscript, was the fact that our participants were USA residents. Given the conflicting nature of the literature around MTurk samples (see Levay et al., 2016; Litman and Robinson, 2021 but also Walters et al., 2018) and the fact that we, ourselves, did not collect detailed sociodemographic information, we have commented, in our limitations, around the generalizability of our results as this is limited by our sample.

“Additionally, since this is an online sample, rather than a random sample from the general population, we caution on inferences about the generalizability of our findings.”

In order to make sure that participants accessed our experiment only once, a qualification was granted to each participant upon completion of our experiment. This is a function of MTurk and is based on each participant’s unique id assigned by MTurk . We then excluded all participants with that qualification from taking part in future experiments.

-*The fact that participants were instructed that the number of points during the task was unrelated to the final compensation limits the ecological validity of this experimental work. The authors should discuss how this aspect might have influenced behavioral responses.*

We thank the reviewer for this comment. In our task, all participants that completed the task, were compensated the same amount of money that was unrelated to their task performance. As we explain in our manuscript, this decision was based on the fact that we wanted participants to input their true momentary mood in response to the task stimuli and not exploit the task in order to obtain maximum gain. Concerning the ecological validity of our task, we believe that such a design closely relates to several real life situations where the final outcome (gain or loss) is unrelated to task performance, for example playing a board game.

-*The authors detail that task exploiters were excluded. Did this group differ from the remaining sample in terms of sex, age, etc.? One might argue that “task exploiters” were individuals who understood the contingency between their ratings and outcome and responded accordingly.*

We have now examined the demographic characteristics of the group of participants that were identified as task exploiters (N:37, age_mean:41, age_std:9, Females:62%), and compared it to the rest of the participants that did not exploit the task (N:150, age_mean:41, age_std:9, Females:54%). No statistical differences were identified concerning age or sex.

In our final experiment of this first set, we did show that when participants were left without instruction, the vast majority of them understood the contingency and exploited the task. In that perspective, exploiters were individuals who understood the contingency (as the majority of participants) but chose to exploit it, deviating from task instructions.

-*It is unclear how the necessary sample size was determined. Did the authors calculate a priory power calculation? This seems particularly important as it was found that only environments were mood was rewarded showed significant effects in training mood.*

Concerning the power analysis, the second set of our experiments, as mentioned in our manuscript, is an exploratory one and a power calculation was therefore not conducted. For those experiments, we did not focus on studying the task environments *per se,* but rather on the fact that mood could be trained. As a result, we aimed for a statistically significant effect that could be visible even with a relatively small sample size. Given the risk of small sample sizes -mainly type II error but also a risk for type I error (Leppink et al., 2016)- , we replicated our findings (for the first two most effective environments), in the next steps of our study, in a larger sample, and also examined whether this effect could survive over time. Since a power calculation was not conducted for that experiment and a post-hoc power analysis is not considered a valid method to assess power (Dziak et al., 2020), we instead conducted a sensitivity analysis to determine the magnitude of effect we were powered to detect (we have added information about this sensitivity analysis to supplementary material). For the last replication experiment with 121 participants (59 experimental and 62 controls), 3 predictors and alpha of 0.0166 after Bonferroni correction (for 3 terms), we had 80% power to detect an f2 of 0.0743. Since we have been careful not to base our conclusions on the lack of significant results, the primary threat to our interpretation from an underpowered study would be type-s errors (Gelman and Carlin, 2014). An f2 of 0.0743 is considered small (Cohen, 1998; Selya et al., 2012) and reasonable to observe for an intervention modifying the response of mood. As such, we do not think a type-s error is likely. Additionally, the statistical power of that analysis is also reflected on the confidence intervals (CIs) of our results (Hoenig and Heisey, 2001; Schulz and Grimes, 2005; Vandenbroucke, 2007), in the task environment which yielded positive results and survived Bonferroni correction. To summarize, the main question addressed in our manuscript is whether mood can be trained which means that one positive finding is sufficient. As such, for the environment that had the largest effect, two more repetitions were performed in novel and larger samples that yielded statistically significant results. Moreover a sensitivity analysis for the final experiment was conducted which showed that it had the power to detect a small effect size. Given that, a type-I or type-s error seems unlikely. Since our main question or interpretation is not based on statistically non-significant results type-II errors, although possible for the remaining environments, do not affect our study.Our sensitivity analysis is now included in our Supplementary Material.

*-As the authors relied on a self-report measure of mood, it would have been beneficial to collect indices of social desirability and control for this aspect.*

We agree with the reviewer that as our experiments are based on subjective mood ratings, social desirability could indeed have an effect on our task findings. However, the testing sequence was provided before (test) and after (retest) our intervention, i.e. within subjects, in order to eliminate these inter-individual differences, including social desirability, that could impact our findings.

*-Table 1: Please report means and SDs with one decimal place.
-Results: Please report <.001 instead of e.g. p<.00001 or p<000019.*

We thank the reviewer for pointing these out. We have now implemented their suggestions for our Table and Results section.

Discussion

*-The authors might want to discuss that future studies would benefit from also collecting objective indices of mood (e.g. psychophysiological parameters) instead of only relying on self-report.*

We thank the reviewer for this comment as it touches upon one of the major questions in psychiatry: How to objectively assess symptoms and mood? The majority of psychiatric assessments are currently based on subjective reports. Although this matter is beyond the scope of our manuscript, we have added a mention of the objective measures that could help assess mood in our future steps.

“Finally, it is standard practice, for psychiatric assessments as well as the majority of studies that examine mood, to only rely on subjective mood ratings. Future studies could complement subjective mood measures with other assessments, including physiological metrics (heart rate, pupillometry), that could help further characterize mood and mood resilience to negative stimuli.”

*-The authors highlight that participants were not required to perform any action to receive or lose points. To what extent might this have influenced the results? It is unclear why the authors took this approach that differs from previous research. Please justify and provide a rationale in the introduction.*

We thank the reviewer for pointing this out. Our task was designed to examine whether mood could be trained by the outcomes of the task environments.t Action is usually used to examine the effects that different aspects of decision making (ex reward prediction error) could have on mood. If our task included decisions (actions), then mood would have been affected not only by the outcomes, which we aim to investigate, but also by various decision-related effects, including the effort required to perform the task actions, or regrets/satisfaction of previous decisions. This is why, in our task, our participants were gaining or loosing points without performing any action. Additionally, this absence of action, entails a fair degree of ecological validity as many of the stimuli that influence our mood, for example, weather, good and bad news, accidents, do not require any action from our part. We have now explained our rationale in our manuscript’s Introduction.

“Since the task focused on mood training by the outcomes of task environments and not on the examination of the effects that different aspects of decision making (ex reward prediction error) could have on mood, no action is required from the participants and stimuli are automatically administered. Such a setting allows to examine the direct effect of task outcomes on mood without any decision related effect such as effort required to perform the task actions, or regrets/satisfaction of previous decisions. Moreover, it could be paralleled to real life events that impact our mood and do not require any action from the individual’s behalf, including the weather, accidents, good and bad news.”

*Additional comments:
-Table 1: …in these rounds is calculated (instead of calculate)*

“We thank the reviewer. It is now corrected”

Sincerely,

Vasilios Mantas, MD, PhD

Vasileia Kotoula, PhD

Relevant Bibliography

- Cohen J. E. (1988). Statistical Power Analysis for the Behavioral Sciences. Hillsdale, NJ: Lawrence Erlbaum Associates, Inc
- Dziak, J.J., Dierker, L.C., Abar, B., 2020. The interpretation of statistical power after the data have been gathered. Curr. Psychol. 39, 870–877. https://doi.org/10.1007/s12144-018-0018-1
- Gelman, A., & Carlin, J. (2014). Beyond Power Calculations: Assessing Type S (Sign) and Type M (Magnitude) Errors. Perspectives on Psychological Science, 9(6), 641–651. https://doi.org/10.1177/1745691614551642
- Hoenig, J.M., Heisey, D.M., 2001. The Abuse of Power: The Pervasive Fallacy of Power Calculations for Data Analysis. Am. Stat. 55, 19–24. https://doi.org/10.1198/000313001300339897
- Keren, H., Zheng, C., Jangraw, D.C., Chang, K., Vitale, A., Rutledge, R.B., Pereira, F., Nielson, D.M., Stringaris, A., 2021. The temporal representation of experience in subjective mood. eLife 10, e62051. https://doi.org/10.7554/eLife.62051
- Levay, K.E., Freese, J., Druckman, J.N., 2016. The Demographic and Political Composition of Mechanical Turk Samples. SAGE Open 6, 215824401663643. https://doi.org/10.1177/2158244016636433
- Leppink, J., Winston, K. & O’Sullivan, P. Statistical significance does not imply a real effect. Perspect Med Educ 5, 122–124 (2016). https://doi.org/10.1007/s40037-016-0256-6
- Litman, L., Robinson, J. (Eds.), 2021. Conducting online research on Amazon Mechanical Turk and beyond, SAGE innovations in research methods. SAGE, Los Angeles.
- Rutledge, R.B., Skandali, N., Dayan, P., Dolan, R.J., 2014. A computational and neural model of momentary subjective well-being. Proc. Natl. Acad. Sci. 111, 12252–12257. https://doi.org/10.1073/pnas.1407535111
- Schulz, K.F., Grimes, D.A., 2005. Sample size calculations in randomised trials: mandatory and mystical. The Lancet 365, 1348–1353. https://doi.org/10.1016/S0140-6736(05)61034-3
- Vandenbroucke, J.P., 2007. Strengthening the Reporting of Observational Studies in Epidemiology (STROBE): Explanation and Elaboration. Ann. Intern. Med. 147, W. https://doi.org/10.7326/0003-4819-147-8-200710160-00010-w1
- Walters, K., Christakis, D.A., Wright, D.R., 2018. Are Mechanical Turk worker samples representative of health status and health behaviors in the U.S.? PLOS ONE 13, e0198835. https://doi.org/10.1371/journal.pone.0198835
- Selya AS, Rose JS, Dierker LC, Hedeker D, Mermelstein RJ. A Practical Guide to Calculating Cohen's f(2), a Measure of Local Effect Size, from PROC MIXED. Front Psychol. 2012 Apr 17;3:111. doi: 10.3389/fpsyg.2012.00111. PMID: 22529829; PMCID: PMC3328081.
